# Genomic Signatures of Island Colonisation in Highly Diverse Primates

**DOI:** 10.1111/mec.17815

**Published:** 2025-07-09

**Authors:** I. Colmonero‐Costeira, K. Guschanski, S. L. Djaló, N. Fernandes, T. Camará, K. K.‐H. Farh, L. F. K. Kuderna, J. Rogers, T. Marques‐Bonet, M. W. Bruford, I. M. Russo, A. Jensen, M. J. Ferreira da Silva

**Affiliations:** ^1^ School of Biosciences Cardiff University Cardiff Wales UK; ^2^ CIBIO—Centro de Investigação em Biodiversidade e Recursos Genéticos, InBIO, Laboratório Associado Universidade do Porto Vairão Portugal; ^3^ BIOPOLIS—Program in Genomics, Biodiversity and Land Planning CIBIO Vairão Portugal; ^4^ CIAS—Research Centre for Anthropology and Health, Department of Life Sciences Universidade de Coimbra Coimbra Portugal; ^5^ Department of Ecology and Genetics, Animal Ecology Uppsala University Uppsala Sweden; ^6^ Institute of Ecology and Evolution, School of Biological Sciences University of Edinburgh Edinburgh UK; ^7^ Tabanka de Abu Ganogo Guinea‐Bissau; ^8^ Tabanka de Anghôr Orango Guinea‐Bissau; ^9^ Cacine Tombali Guinea‐Bissau; ^10^ Illumina Artificial Intelligence Laboratory Illumina San Diego California USA; ^11^ Human Genome Sequencing Center and Department of Molecular and Human Genetics Baylor College of Medicine Houston Texas USA; ^12^ Institute of Evolutionary Biology (UPF‐CSIC), PRBB Barcelona Spain; ^13^ Catalan Institution of Research and Advanced Studies (ICREA) Barcelona Spain; ^14^ CNAG, Centro Nacional de Analisis Genomico Barcelona Institute of Science and Technology (BIST) Barcelona Spain; ^15^ Institut Català de Paleontologia Miquel Crusafont Universitat Autònoma de Barcelona Cerdanyola del Vallès Barcelona Spain

**Keywords:** Cercopithecini, conservation genomics, genetic load, Guinea‐Bissau, intraspecific variation

## Abstract

Understanding how small populations cope with loss of genetic diversity and deleterious variation is crucial to address the current biodiversity crisis. Insular populations are particularly interesting as they have often persisted at lower population sizes and higher inbreeding than their mainland counterparts. While the genome‐wide consequences of inbreeding in threatened insular species have received some attention, comparative genomics between insular and mainland populations of widespread and genetically diverse species have rarely been performed. Yet, they are particularly well suited to inform about the consequences of drastic population declines from initially large populations—a phenomenon that is becoming increasingly common. The spot‐nosed monkey ( *Cercopithecus petaurista* ), the Campbell's monkey ( *Cercopithecus campbelli* ) and the green monkey ( *Chlorocebus sabaeus* ) are common and genetically diverse West African primates. Insular populations can be found at the Bijagós Archipelago, Guinea‐Bissau. Here, we assessed the genome‐wide diversity, inbreeding, genetic load and adaptive variation using whole genome sequencing data from insular and mainland populations. In the three species, island populations showed lower genome‐wide diversity and higher inbreeding. Genetic drift has likely promoted the conversion of masked genetic load into realised load without increased purging of deleterious variation. Additionally, we found no evidence for accumulation of deleterious variation, suggesting that these populations are not yet at risk of extinction by genetic factors and may act as reservoirs of extant mainland genetic diversity. We highlight, however, that other anthropogenic factors are threatening these insular primates, and therefore conservation management should target their immediate threats and safeguard against additional loss of diversity.

## 1 Introduction

In a time when anthropogenic activities increasingly impact ecosystems, conservation of biodiversity has become a global challenge. Intraspecific genetic diversity has recently received wider recognition by governmental bodies (Convention on Biological Diversity (CBD) 2020; Hoban et al. 2021; Mastretta‐Yanes et al. 2024). Since its inclusion in the CBD's Kunming‐Montreal Global Biodiversity Framework, greater accessibility to genomic data for non‐model species has improved our capacity to disentangle neutral and adaptive components of genetic diversity, opening the door to better understand species demographic and adaptive responses to environmental or anthropogenic changes (Allendorf et al. 2010).

Insular populations can be particularly vulnerable to extinction due to genetic factors resulting from the demographic processes inherent to the colonisation dynamics. Island colonisation typically involves a small number of founding individuals, which carry only a fraction of the total genetic diversity of the source populations (Allendorf et al. 2013; Martin et al. 2023). The intrinsic low levels of genetic diversity in insular populations tend to be aggravated by restricted or no gene flow with the source populations and increased inbreeding among founding individuals and their descendants. Genetic drift is stronger in populations with small effective population size (*N*_e_), as often is the case in insular systems (Allendorf et al. 2013). Although genetic drift is a neutral evolutionary process, it can also incur fitness costs by increasing the allele frequencies of loci with deleterious variation (Bertorelle et al. 2022; Dussex et al. 2023). As masked genetic load (i.e., recessive deleterious mutations present in their heterozygous state) is converted into realised load (i.e., homozygous deleterious mutations with expressed fitness effects; Bertorelle et al. 2022), individual genetic load may increase, reducing the individual fitness and increasing the risk of inbreeding depression (Bertorelle et al. 2022; Dussex et al. 2023; Smeds and Ellegren 2023). However, the increased frequency of strongly deleterious mutations in their homozygous states makes them visible to selection, allowing for their removal through purging (Dussex et al. 2021; Grossen et al. 2020; Khan et al. 2021; van der Valk et al. 2024; von Seth et al. 2022; Xue et al. 2015). Although purifying selection is expected to purge mutations with large fitness effects, strong genetic drift can reduce the efficiency of selection in small populations, potentiating the gradual accumulation of mildly deleterious mutations during prolonged bottlenecks (Dussex et al. 2023). Such accumulation can generate a decline in fitness and exacerbate the extinction risk of small, isolated populations (Bataillon and Kirkpatrick 2000; Khan et al. 2021).

Insular populations frequently exhibit genetic and phenotypic differences compared to mainland populations in response to novel ecosystems. This phenomenon, collectively known as the ‘island syndrome’, often includes changes in morphology, behaviour and life‐history traits (Adler and Levins 1994). It has been attributed to adaptive processes in response to the new habitats where different traits, more advantageous to the insular environment, are selected for (Martin et al. 2021; Payseur and Jing 2021; Sendell‐Price et al. 2020; Welles and Dlugosch 2019). Another hypothesis is that loci under strong selection on the mainland become effectively neutral on the islands due to the relaxation of natural selection (Cui et al. 2021; Wang et al. 2023). Consequently, non‐synonymous variants can drift towards fixation in the insular populations.

Due to their vulnerability coupled with high potential for neutral and adaptive differentiation, insular populations are of great conservation concern. Previous studies have focused mainly on highly threatened insular populations from already genetically depauperate species (e.g., kākāpō, *Strigops habroptilus* ; Dussex et al. 2021; Chatham Island black robin, *Petroica traversi* ; von Seth et al. 2022). However, little attention has been given to widespread and genetically diverse species. Such species, many of which are classified as non‐threatened on the International Union for Conservation of Nature (IUCN) Red List, are becoming increasingly threatened due to anthropogenic activities in their ranges (Finn et al. 2023). As the genomic consequences of inbreeding and decreasing population sizes of widespread populations may differ from those of more threatened taxa (Robinson et al. 2016; Taylor et al. 2024), it is increasingly important to consider non‐flagship species in the field of conservation genomics in order to pre‐emptively promote their conservation.

Here, we investigated the effects of insularity and low population size in three guenon species, tribe Cercopithecini, one of the most genetically diverse African primate groups (Jensen et al. 2023; Kuderna et al. 2023). We used sequencing data to compare genome‐wide diversity, inbreeding and genetic load between insular and mainland populations of the spot‐nosed monkey ( *Cercopithecus petaurista* , Scheber, 1774), the Campbell's monkey ( *Cercopithecus campbelli* , Waterhouse, 1838), and the green monkey ( *Chlorocebus sabaeus* , Linnaeus, 1766) in Guinea‐Bissau, West Africa. Although classified as non‐threatened by the IUCN, these species are declining throughout their ranges (Gonedelé Bi et al. 2020; Matsuda Goodwin, Gonedelé Bi, et al. 2020; Matsuda Goodwin, Segniagbeto, et al. 2020). In Guinea‐Bissau, these are the most hunted primate species for bushmeat trade (Ferreira da Silva et al. 2021; Minhós et al. 2013) and the insular populations are not considered for conservation management by local authorities (Colmonero‐Costeira et al. 2023).

## 2 Methods

### 2.1 Study Area and Focal Species

The Bijagós Archipelago is a continental archipelago of 88 islands and islets located off the coast of Guinea‐Bissau, West Africa. It has been proposed that the separation of the archipelago from the mainland started as early as ca. 19,000 years ago (YA) but is thought to have become fully isolated during the Holocene, particularly during the lower Flandrian transgression, ca. 12,000–6000 YA (Alves 2007). Both climate and vegetation are similar to southern areas of the mainland (Catarino et al. 2008), which is characterised as a mosaic of forest of Guinean affinity and woodland savannah. Non‐human primates coexist with local *Bijagó* communities whose arrival on the archipelago has been associated with the expansion of West African trading empires, including the peak expansion of the Ghana and Mali Empires, ca. 1200–800 YA (Rodney 1970 in Lundy 2015).

The spot‐nosed monkey and the Campbell's monkey are classified by the IUCN as Near Threatened (Matsuda Goodwin, Gonedelé Bi, et al. 2020; Matsuda Goodwin, Segniagbeto, et al. 2020), whereas the green monkey is listed as Least Concern (Gonedelé Bi et al. 2020). These guenon species are the only non‐human primates occurring on the Bijagós Archipelago (Colmonero‐Costeira et al. 2019; Gippoliti and Dell'Omo 2003). Across the archipelago, the distribution of the three species does not overlap except on Caravela Island, where both the spot‐nosed monkey and the Campbell's monkey can be found in sympatry (Colmonero‐Costeira et al. 2019; Gippoliti and Dell'Omo 2003). The three species are widespread and considered habitat generalists, exhibiting high behavioural and dietary flexibility (Bersacola et al. 2022; Rowe and Myers 2016).

In Guinea‐Bissau, the spot‐nosed monkey (*santcu naris‐branku* or *santcu bidjugu* in Guinea‐Bissau Kriol) is possibly restricted to the Bijagós Archipelago (Gippoliti and Dell'Omo 2003). Historically, the species is thought to have been present on the mainland (Gippoliti and Dell'Omo 2003) but has been absent/not reported from the mainland for the last 30 years (Bersacola et al. 2018, 2022; Gippoliti and Dell'Omo 2003; Colmonero‐Costeira, *personal communication*). The Campbell's monkey (*santcu mona* in Guinea‐Bissau Kriol) and the green monkey (*santcu di tarafe* in Guinea‐Bissau Kriol) are considered the most abundant non‐human primate species in the country (Bersacola et al. 2018; Karibuhoye 2004; Gippoliti and Dell'Omo 2003). However, past studies suggest that their populations are threatened by illegal commercial hunting and habitat destruction (Gippoliti and Dell'Omo 2003). Campbell's and green monkeys are the most traded primates for meat consumption in the urban bushmeat markets in the capital city and the most commonly available species in restaurants/bars in the south of the country (Ferreira da Silva et al. 2021; Minhós et al. 2013). On the Bijagós Archipelago, the commercial trade of spot‐nosed monkey meat has been recently described (Colmonero‐Costeira et al. 2023) and subsistence hunting is reported for the remaining two species (see below).

### 2.2 Sample Collection

Tissue samples of spot‐nosed monkeys, Campbell's monkeys, and green monkeys were obtained as part of previous research that focused on primate meat hunting and consumption in Guinea‐Bissau between 2010 and 2017. Samples from the Bijagós Archipelago were collected directly from local hunters or villagers preparing the carcasses for consumption (2002–2016). Samples from mainland Guinea‐Bissau were collected at meat markets in the capital city, Bissau (March—June 2010; Minhós et al. 2013), or from drinking establishments located in the vicinity of protected areas in southern mainland (2015–2017; Ferreira da Silva et al. 2021). The DNA extractions of samples collected in Bissau is described in Minhós et al. (2013). For the remaining samples, we extracted DNA using a salting out procedure (Miller et al. 1988) at the CIBIO‐InBIO research centre, Portugal. Negative controls were included throughout the entire process to test for DNA contamination. Samples with low DNA concentration were re‐extracted using the Qiagen DNeasy Blood & Tissue kit (QIAGEN Hilden, Germany) following the manufacturer's protocol. To minimise the inclusion of highly related individuals in the dataset, we selected samples collected in different weeks, different commercial establishments or different hunting events. A total of 50 samples were sequenced on Illumina NovaSeq6000 (150 bp PE), aiming for 30× coverage per sample. We generated high quality whole‐genome sequences from the three guenon species present on the Bijagós Archipelago (eight spot‐nosed, one Campbell's and three green monkeys), and their mainland counterparts of Campbell's (*N* = 25) and green monkey (*N* = 10) (Supporting Information S2). Since it is likely that no spot‐nosed monkeys are present on the mainland Guinea Bissau, we included a publicly available spot‐nosed monkey genome of unknown geographic origin from mainland Africa in the analyses (Kuderna et al. 2023).

### 2.3 Mapping and Variant Calling

We used Picard v2.23.4 (http://broadinstitute.github.io/picard/) to add read group information and mark adapter content in the sequencing reads. Reads were aligned to the rhesus macaque ( *Macaca mulatta* ) reference genome (Mmul_10, GCF_003339765.1) with the Burrows‐Wheeler Aligner (BWA) v0.7.17 (Li and Durbin 2009). Picard was used to sort and deduplicate the mapped bam‐files. Next, we used GATK v4.3.0.0 to call genotypes per sample with HaplotypeCaller, which were subsequently combined with CombineGVCFs and jointly genotyped with GenotypeGVCFs. We excluded insertions and deletions, and used VariantFiltration to filter variants based on the following exclusion criteria: QD < 2.0, QUAL < 30.0, SOR > 3.0, FS > 60.0, MQ < 40.0, MQRankSum < −12.5, ReadPosRankSum < −8.0. Repetitive regions were excluded following the SNPable regions pipeline (https://lh3lh3.users.sourceforge.net/snpable.shtml). We also masked heterozygous genotypes with skewed allelic balance (minor allele support < 0.25), as well as genotypes with less than half or more than double the genome‐wide average read depth per sample.

### 2.4 Mitochondrial Genome Assembly and Haplotype Networks

The mitochondrial genomes were assembled using the MitoFinder pipeline (Allio et al. 2020). We started the assembly by trimming adapter sequences using Trimmomatic (Bolger et al. 2014) and running MitoFinder with the metaspades assembler. The assembled contigs were corrected for circularization and rotated to the same starting position using a custom Python script (https://github.com/axeljen/guenon_phylogenomics) and annotated using the green monkey (NC_008066.1) reference mitochondrial genome. Annotated assemblies were aligned for each species separately using MAFFT aligner (Katoh et al. 2002). We inspected the species‐specific alignments visually and trimmed the non‐coding hypervariable region 1 (positions 16,010—16,361) and hypervariable region 2 (positions 56–364) of the green monkey reference genome and misassembles/misalignments. For the spot‐nosed monkey, we included publicly available mitochondrial genomes (JQ256931.1, JQ256932.1, JQ256982.1 and JQ256983.1) in the final alignment (Guschanski et al. 2013). We visualised the relationships between the assembled mitochondrial genomes by reconstructing median joining haplotype networks using PopART (Leigh and Bryant 2015).

### 2.5 Population Structure, Divergence Times and Effective Population Size

To assess population structure between the insular and mainland populations, we conducted a principal component analysis (PCA) in PLINK v1.9 (Purcell et al. 2007), removing positions with missing genotypes within each species (‐‐*geno 0*) and thinning the total autosomal SNPs to 100,000. We inferred the demographic history of the populations using three complementary methods: the pairwise sequentially Markovian coalescent model implemented in beta‐PSMC (Liu et al. 2022), the multispecies coalescent model implemented in BPP (Flouri et al. 2018), and demographic modelling based on linkage disequilibrium (LD) between pairs of loci as implemented in GONE (Santiago et al. 2020). We ran beta‐PSMC (Liu et al. 2022) with default parameters and five sub‐atomic time intervals. The variance of the estimated *N*_e_ trajectories was assessed by 20 randomised resampling runs across the genome. We scaled the output assuming a mutation rate of 4.65 × 10^−9^ per site per generation (Kuderna et al. 2023) and a generation time of 11 years for the spot‐nosed monkey and 12 years for the Campbell's monkey and green monkey (Gonedelé Bi et al. 2020; Matsuda Goodwin, Gonedelé Bi, et al. 2020; Matsuda Goodwin, Segniagbeto, et al. 2020). Beta‐PSMC was chosen over the PSMC method (Li and Durbin 2011) as it provides a more detailed and accurate estimation of population size changes at more recent timescales (Liu et al. 2022), and is less sensitive to artefacts commonly reported in PSMC (Hilgers et al. 2025). We used BPP to estimate divergence times and effective population sizes (*N*_e_) of mainland and island guenon populations. Due to lack of genetic differentiation (see below), green monkeys from Ganogo and Orango islands were considered as one population in these analyses (OIG, Orango Island Group), and we used a single representative per population (the sample with the highest sequencing coverage). We randomly sampled autosomal loci of 1 kb each, located at least 10 kb from the nearest gene and at least 50 kb from each other, requiring at least 800 bp without missing data. We first ran bpp to estimate the species tree (*speciestree* = 1), confirming that the island populations of the spot‐nosed monkeys were monophyletic. Next, we ran two independent analyses to estimate divergence times and effective population sizes. We discarded the first 2000 MCMC iterations as burnin, and then sampled every second iteration until 200,000 samples were obtained. After confirming that the two runs converged at highly similar parameter estimates, the two runs were merged and jointly summarised using the bppr package v0.6.3 (Reis 2025). We used the same mutation rate used in the bPSMC analyses (4.65 × 10^−9^) and a generation time of 11 years to scale the theta and tau estimates to effective population size and geological time, respectively. For the spot‐nosed monkeys of Canhabaque Island (*N* = 7), we inferred more recent changes in *N*_e_ using GONE. This species was chosen as a representative of the island populations for its sufficiently large sample size, permitting the use of this method. We used the default parameters, aside from excluding SNP positions for which at least one individual was not genotyped (*ZERO* = 0). We used the rhesus macaque ( *Macaca mulatta* ) average genome‐wide recombination rate of 0.448 cM/Mb (Xue et al. 2020). To incorporate variance in the estimated *N*_e_ trajectories, GONE was run 20 independent times using a random subset of 2,000,000 SNPs generated using PLINK.

### 2.6 Genome‐Wide Diversity and Inbreeding

We identified autosomal runs of homozygosity (ROH) using PLINK v1.9 and estimated autosomal heterozygosity for each individual genome using vcftools (Danecek et al. 2011). To minimise the detection of false positive ROH, we scaled the number of SNPs that constituted a ROH (‐‐*homozyg‐snp*) by the within species average heterozygosity (Purfield et al. 2012):

$$L=\frac{\log_{e}\frac{a}{n_{s}n_{i}}}{\log_{e}\left(1-\mathrm{het}\right)}$$

where *n*_s_ is the number of genotyped SNPs, *n*_i_ the number of genotyped individuals, *α* the false positive ROH threshold (set at 0.05), and *het* the mean heterozygosity. Based on *L*, the minimum number of SNPs that constituted a ROH was set to 77, 88, and 81 for the spot‐nosed monkey, the Campbell's monkey and the green monkey, respectively. We selected a scanning window length equal to *L* as suggested by (Meyermans et al. 2020). For the remaining ROH search parameters, we used the default values. The fraction of the genome contained in ROH (*F*_ROH_) for each individual was estimated as:

$$F_{\mathrm{ROH}}=\frac{L_{\mathrm{ROH}}}{L_{\mathrm{ROH},\mathrm{HOM}}}$$

where *L*_ROH_ is the sum of the length of all detected ROHs, and *L*_ROH,HOM_ is the maximal detectable ROH length of a synthetic homozygous genome of each individual, under the same ROH detection parameters (Meyermans et al. 2020). The synthetic homozygous genome for each individual was obtained by changing all heterozygous genotypes to homozygous using a custom Python script. This minimised the impact of stochastic genome‐specific sequencing artefacts that potentially prevent the correct detection of ROH in window‐based methods (Meyermans et al. 2020).

We estimated the distribution of ROHs arising from inbreeding after the isolation of the Bjagós Archipelago or during human coexistence using the expected ROH coalescent time in generations (*g*):

$$g=\frac{100}{2\mathrm{rl}}$$

Equivalent to:

$$l=\frac{50}{\mathrm{rg}}$$

where *r* is recombination rate and *l* the ROH length (Thompson 2013). We used the rhesus macaque average genome‐wide recombination rate of 0.448 cM/Mb. As such, we established ROH ≥ 1 Mb as human‐contemporary (up to 110 guenon generations, ≈ 1200 YA) and ROH < 1 Mb as historical (older than 110 guenon generations ago). The chosen time windows reflect the earliest time of the arrival of humans to the Bijagós Archipelago and the isolation of the archipelago, respectively.

### 2.7 Genetic Load

To investigate if isolation on the islands may have led to the purging of deleterious mutations, we characterised the derived positions in coding regions across the insular and mainland guenon genomes using Ensembl's variant effect predictor (VEP; McLaren et al. 2016). Prior to the VEP annotation, we polarised the ancestral alleles using the Guinea baboon ( *Papio papio* ; GCA_028645565.1), the Angolan colobus monkey ( *Colobus angolensis* ; GCF_000951035.1), and rhesus macaque (Mmul_10) as outgroups. The additional outgroups were aligned to the Mmul_10 reference genome using Minimap2's cross‐species full‐genome alignment (Li 2018), assuming a species divergence below 20% (*asm20*). We called the genotypes on the outgroups using bcftools mpileup, multiallelic‐caller (−*m*) (Danecek et al. 2021). Alleles present in the three outgroup species in the homozygous state were defined as ancestral. These positions were identified using a custom Python script. Invariable sites in the guenons were removed for downstream analysis.

The effects of the retained variants were predicted based on the rhesus macaque (Mmul_10) annotations. For sites on overlapping transcripts, we selected a single prediction per variant based on VEP's standard ordered set of criteria (‐‐*pick*), including the canonical status of a transcript, APPRIS functional isoform notation (most functionally important transcripts), transcript support level, biotype (protein coding preferred), consequence rank (most severe). We classified the annotated variants as loss‐of‐function (LoF: transcript ablation, stop gained, stop lost, start lost, frameshift variant, splice region variant, splice donor variant and splice acceptor variant), missense and synonymous. Missense variants were further classified into two classes: (1) likely deleterious or (2) tolerated, using the Experimental exchangeability score of amino acids (EX; Yampolsky and Stoltzfus 2005). We calculated the EX of missense variants using a custom Python script adapted from Smeds and Ellegren (2023) (https://github.com/linneas/wolf‐deleterious). Sites were classified as likely deleterious if the Experimental exchangeability score was ≤ 0.256 (Williamson et al. 2005).

A decrease in the number of deleterious variants across the genome can potentially arise as a combination of purifying selection and stochastic loss due to genetic drift. Considering that without selection, genetic drift does not change allele counts of the derived mutations (Dussex et al. 2023), we estimated the individual genetic load as the relative abundance (RA) of the derived alleles (*A*) for each individual (*i*) of population *x* and standardised it by the number of derived alleles within species across the different mutational categories:

$${\mathrm{RA}}_{A\;\mathrm{ix}}=\frac{n_{R/A\;i}+2n_{A/A\;i}}{2n_{p}}$$

where *n*_R/A i_ is the number of derived heterozygous positions (*R*, reference allele; *A*, derived allele), *n*_A/A I_ is the number of derived homozygous positions, and *n*_p_ is the total number of derived positions across all the genomes for a particular species (insular and mainland).

Furthermore, based on the VEP output, we performed a functional analysis of the LoF variants that have become fixed in the homozygous state in insular populations. We conducted a gene ontology (GO) enrichment analysis using g:Profiler (https://biit.cs.ut.ee/gprofiler/gost) and the g:SCS multiple testing correction algorithm on these sets of genes (Kolberg et al. 2023).

### 2.8 Differential Variation in Protein‐Coding Genes

We identified variants in protein‐coding regions that were fixed for alternative alleles between insular and mainland populations of the same species using a custom Python script (Jensen et al. 2023). We annotated the differentially fixed positions using VEP. To filter out variants that accumulate through drift in potential pseudogenes, we removed all genes that contained LoF variants. Next, we looked for sets of genes that contained differentially fixed variants in at least two of the studied guenon species and conducted a GO enrichment analysis using g:Profiler.

## 3 Results

### 3.1 Sequencing and Genotyping

We successfully generated high quality whole‐genome sequences for 48 samples. We found two mainland Cambell's monkeys and one mainland green monkey were likely sampled twice. The repeat samples were excluded from the analyses, keeping one sample per individual. The final dataset consisted of 44 newly generated whole‐genome sequences (eight spot‐nosed, 24 Campbell's and 12 green monkeys). Average autosomal mapping coverage of the dataset varied between 25.95× and 35.00× (average = 30.32×; Supporting Information S1). After calling the genotypes against the rhesus macaque Mmul_10 reference genome, we identified 18,415,827 autosomal biallelic SNPs in the spot‐nosed monkey, 21,836,509 SNPs in the Campbell's monkey, and 15,716,160 SNPs in the green monkey. Additionally, we assembled and annotated the mitochondrial genomes for all the samples.

#### 3.1.1 The Three Insular Populations Are Differentiated From Mainland Populations and Show Reduced Long‐Term *N*_e_

**FIGURE 1:**
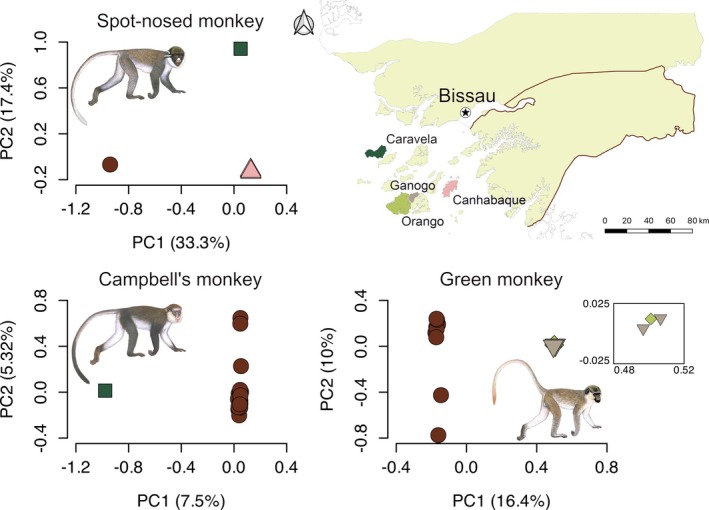
Principal component analysis (PCA) based on autosomal biallelic single nucleotide polymorphisms of insular and mainland guenons of Guinea‐Bissau. An inset focusing on the insular green monkeys is shown on the right of the green monkey PCA plot. Each individual sample is coloured according to their sampling area, as illustrated on the map of Guinea‐Bissau, West Africa. The exact provenance of the mainland samples of Campbell's and green monkeys is not known but they likely originate from Southern Guinea‐Bissau (represented by the brown contour in the map in the upper right side) (Minhós et al. 2013; Ferreira da Silva et al. 2021). The geographic origin of the mainland spot‐nosed sample is unknown, but it likely belongs to the eastern subspecies based on mtDNA analyses (Figure S2). Illustrations copyright 2022 Stephen D. Nash/IUCN SSC Primate Specialist Group. Used with permission.

The PCAs suggested the existence of population structure in all three guenon species, with PC1 differentiating insular and mainland populations within each species and explaining 33.3%, 7.5% and 16.4% of the variance for the spot‐nosed, Campbell's and green monkeys, respectively (Figure 1). Further differentiation along PC2 was observed between the spot‐nosed monkey individuals from Canhabaque and Caravela Islands. In contrast, the green monkeys from Ganogo and Orango Islands (separated by a ~500 m water channel) were almost indistinguishable from each other in the PC space. In the absence of clear population structure, individuals from these two neighbouring islands were treated as one population in downstream analyses, designated as the Orango Islands Group (OIG).

None of the mtDNA haplotypes sampled from the islands were shared with the mainland in any of the three species (Figures S1–S4). In the Campbell's monkey and the green monkey, the pairwise number of substitutions between insular and mainland haplotypes was within the range of the pairwise comparisons among the mainland haplotypes. In contrast, the spot‐nosed monkey showed insular haplotypes that differed from each other by only a few substitutions, but these haplotypes were separated from the mainland haplotype by more than 400 substitutions. Since the geographic origin of the mainland spot‐nosed individual is not known, this separation could be the result of deep population structure within the species, such as genetic differentiation at the subspecies level. After the inclusion of the historical mtDNA genomes (Guschanski et al. 2013), the mtDNA haplotype from the mainland spot‐nosed monkey included in the dataset was more closely related to mtDNA haplotypes sampled in Togo, Liberia and Ivory Coast. This result suggests the mainland individual used in this study could be from the eastern *C. petaurista* subspecies ( *C. petaurista petaurista* , Jentink, 1886), whereas the archipelago individuals likely belong to the western subspecies (*C. petaurista buettikoferi*, Jentink, 1886), which would explain the deep mitogenomic divergence.

**FIGURE 2:**
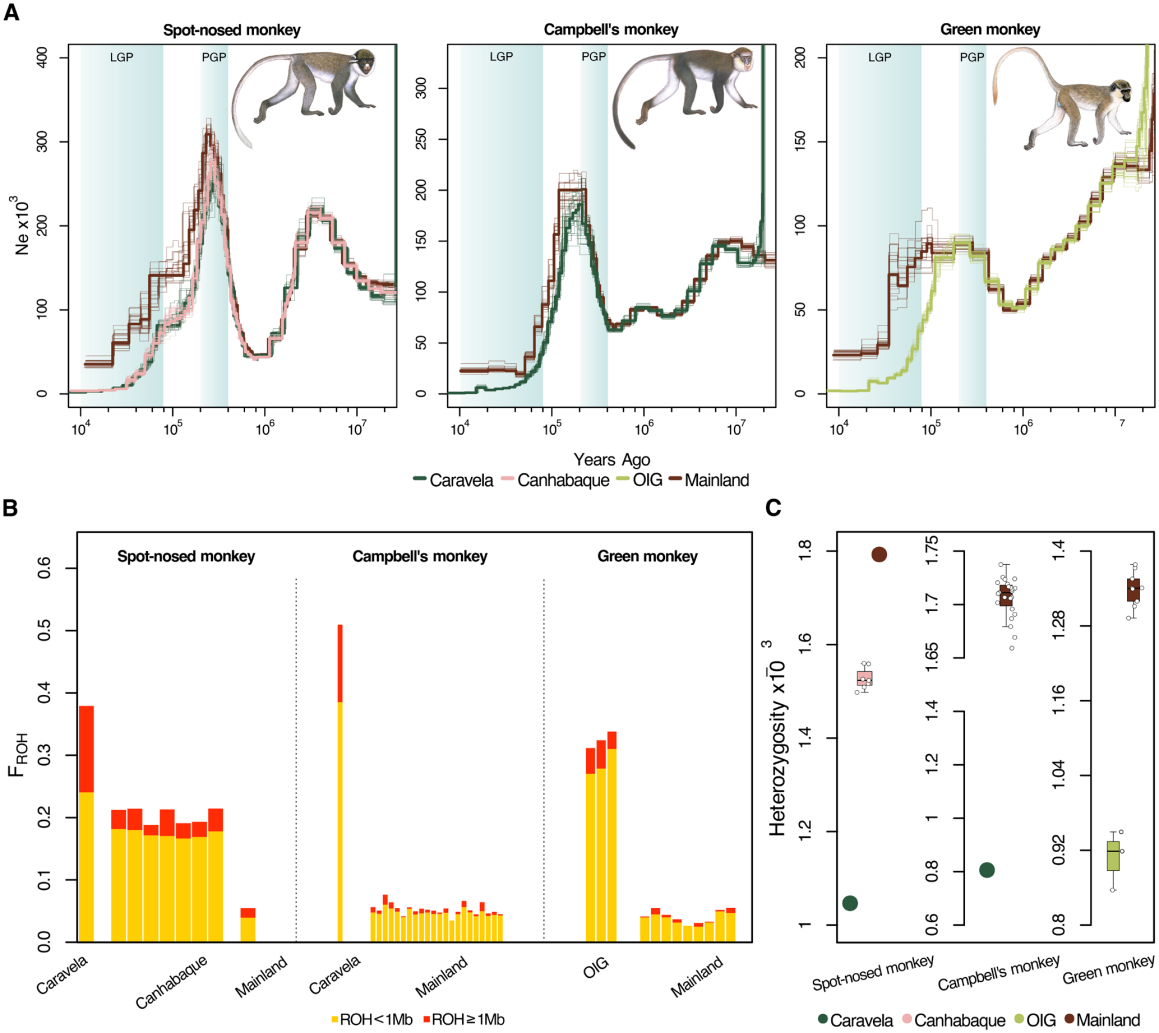
Genome‐wide consequences of insularity in spot‐nosed (left), Campbell's (centre), and green monkey populations (right). (A) Estimation of long‐term changes in *N*_e_ of populations using the pairwise sequentially Markovian coalescent model of insular (Caravela, Canhabaque, Orango Island Group—OIG) and mainland populations as inferred using beta‐PSMC (Liu et al. 2022). Due to the lack of differentiation, green monkeys from Ganogo and Orango islands were considered as one population in these analyses (OIG). Colours correspond to the sampling locations. Bootstrap runs are represented as pale lines. Light blue backgrounds correspond to the Last Glacial Period (LGP) and the Penultimate Glacial Period (PGP). For illustrative purposes, only a single individual per population is represented as the variation within populations is negligible (Figure S5). (B) Fraction of the genome in ROH (*F*_ROH_). *F*_ROH_ for each sampled individual is represented in the stacked bars. The proportion of short ROHs (< 1 Mb, 111–1450 guenon generations, ~16,000–1211 YA) and long ROHs (≥ 1 Mb, 110 generations ago, ~1200 YA) are represented in yellow and red, respectively. (C) Heterozygosity based on bi‐allelic autosomal SNP positions. The circles or boxplots (when *N* > 3 genomes) were coloured according to the sampling location. Differences in autosomal heterozygosity between insular and mainland green monkeys were significant (Welch's two sample *t*‐test, *p* < 0.01; statistical significance could not be tested for the other species [*N* < 3 genomes in one of the groups]). Green monkey individuals from Ganogo and Orango islands were clustered together into the Orango Islands Group (OIG). Note differences on the y‐axis for each species. Illustrations copyright 2022 Stephen D. Nash/IUCN SSC Primate Specialist Group. Used with permission.

We estimated changes in *N*_e_ over time using beta‐PSMC analyses. Overall, the three species showed different demographic histories roughly before the onset of the Penultimate Glacial Period (PGP, > 200,000 YA; Figure 2A). The maximum *N*_e_ of the three species was observed close to the end of the PGP (slight timing variation between species), after which they all underwent a severe decline in *N*_e_ that extends until the end of the Last Glacial Period (LGP, ~12,000 YA). The insular populations showed more rapid and severe declines than the mainland populations since the PGP, with population trajectories diverging between 100,000 and 40,000 YA in the three species. We also inferred the demographic history of the insular and mainland guenon populations using BPP. Island and mainland populations were estimated to have diverged between 600,000 and 160,000 YA, which was accompanied by a drastic decline in *N*_e_ in all insular populations relative to the mainland conspecifics (Figure S6). Effective population size estimates of insular spot‐nosed monkeys were ~18% (Caravela) and ~40% (Canhabaque) of the mainland population *N*_e_. Campbell's and green monkeys were ~7% and 14% of their mainland conspecifics, respectively. Nevertheless, the *N*_e_ estimates of insular guenon populations were in the range of ~10,000 to ~46,000 individuals. Contemporary *N*_e_ estimates of the insular spot‐nosed monkeys from Canhabaque Island (the only insular population with adequate sample size to run GONE analysis) suggested a much smaller population size (~2300 individuals) and a bottleneck‐like signal around 3000 YA (Figure S7).

#### 3.1.2 Insular Populations Show Signatures of Elevated Inbreeding and Loss of Genetic Diversity

The insular individuals of the three species showed ~4–10 times higher proportion of the genome in ROH than mainland populations (Figure 2B). The differences between insular and mainland populations were driven by both an increase in short (< 1 Mb) and long ROHs (≥ 1 Mb). The largest differences in the proportion of the genome in long ROHs were found in the spot‐nosed monkey and Campbell's monkey from Caravela Island, which displayed a ~9 and 71 times increase compared to the mainland individuals of the same species, respectively. Moreover, all insular individuals displayed lower heterozygosity (~15%–53% fewer autosomal heterozygous positions, Figure 2C).

#### 3.1.3 Island Populations Do Not Show Increased Purging nor Accumulation of Deleterious Variants

**FIGURE 3:**
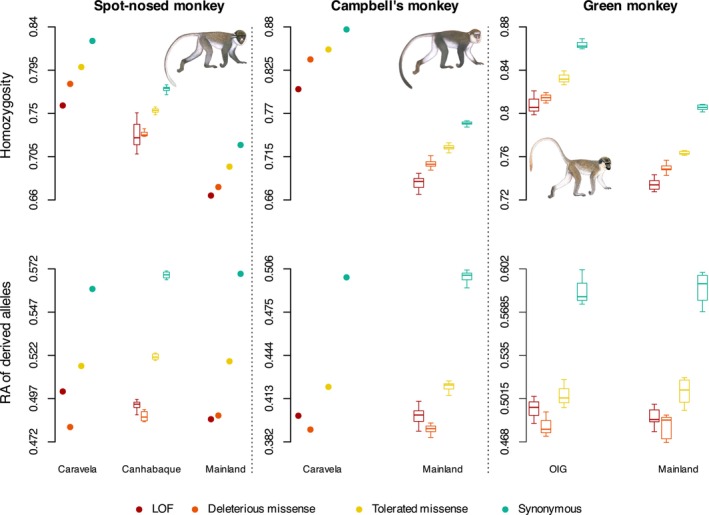
Individual mutational load in insular spot‐nosed (left), Campbell's (centre), and green monkey populations (right). Upper panels: Homozygosity of variants across different categories of mutational effects of the annotated derived SNPs. Lower panels: Relative abundance of derived alleles. Boxplots are presented when *N* > 3 individuals. The statistical comparisons for both the homozygosity and the relative abundance of derived alleles was performed within each variant category (e.g., LoF insular vs. LoF mainland) for the green monkey (Welch's two sample *t*‐test: Homozygosity *p* < 0.01 across all categories; relative abundance of derived alleles, 0.52 < *p* < 0.90 across all categories; statistical significance could not be tested for the other species [*N* < 3 genomes in one of the groups]). Missense mutations were classified as potentially deleterious or tolerated based on the experimental exchangeability score (EX, Yampolsky and Stoltzfus 2005).

To understand if small effective population sizes and increased inbreeding associated with insularity led to changes in the frequency of deleterious mutations, we estimated the individual genetic load for each genome. After identifying the ancestral states of the segregating SNPs in the guenon species using three outgroup species, a total of 181,372 derived SNPs in the spot‐nosed monkey, 217,114 in the Campbell's monkeys, and 188,879 in green monkeys were annotated (Table S1). In general, insular and mainland populations showed similar patterns across all categories of derived variants. In all three species, we found that the proportion of homozygous derived variants is higher in island individuals than in mainland individuals regardless of variant category (Figure 3). This is a pattern consistent with the conversion of masked into realised genetic load in the insular populations by inbreeding and genetic drift. There was a general trend of lower numbers of LoF and other deleterious variants among insular individuals compared to their mainland conspecifics (Table S1). However, after standardising for the relative abundance of segregating alleles within species, there were no apparent differences between islands and mainland individuals (Figure 3). Additionally, we highlight that the patterns between the genetic load of insular and mainland individuals across different categories of deleterious variants were similar to those of synonymous variants, which are expected to behave neutrally (Figure 3).

In the insular spot‐nosed monkeys, we identified a total of 17 genes containing LoF variants that have become fixed in homozygous states, 32 genes in the insular Campbell's monkey, and eight genes in the insular green monkeys (Supporting Information S2). These sets of genes were species‐specific, with some being associated with immune response (spot‐nosed monkey: APOL5; Campbell's monkey: ACP5, ADGRG5, TRBV7‐3, REG3G; green monkey: IFI16) and fecundity (Campbell's monkey: PADI6). Gene ontology (GO) enrichment analysis revealed no overrepresented GO terms.

#### 3.1.4 Differential Variation in Protein‐Coding Genes Between Island and Mainland Populations Is Enriched for Developmental Processes

To explore the hypothesis that insularity can lead to specific, potentially adaptive, genetic and phenotypic changes between insular populations and their mainland counterparts, we identified all differentially fixed positions between the island and mainland populations in each species. We found 2988 differentially fixed variants in protein‐coding genes for the spot‐nosed monkey, 656 for the Campbell's monkey, and 176 for the green monkey. Within these gene sets, 203 genes were found in at least two species (Figure S8). The GO enrichment analysis of this overlapped gene set revealed several overrepresented terms, mostly related to various metabolic functions and organism developmental processes (Supporting Information S3). Specifically, several of the genes with differential variation between insular and mainland populations are involved in cellular division and organogenesis (PDGFRA, TGFB2, E2F8, NIN), neuronal and muscular development (SYNE2, BRINP1, TNC, ARNT2, TENM4, ACTN3, FMN1), skeletal development (CHST11, COL2A1) and thyroid development and functioning (PAX8).

## 4 Discussion

Insular populations are often of conservation concern as they are typically more susceptible to loss of genetic diversity due to demographic processes associated with island colonisation. In this work, we explored the genomic signatures of insularity and reduced population size in guenons, a primate group known for their overall high genetic diversity (Jensen et al. 2023; Kuderna et al. 2023). We compared island and mainland populations in three guenon species from the Bijagos Archipelago, Guinea‐Bissau (in West Africa). In the presence of high sample size skew, as often is the case in opportunistic sampling schemes, and aware of its implications on population‐level statistics, we employed mostly individual‐based statistics and explored the common genetic patterns between the three species which likely represent the broad effects of insularity. Finally, we use our results to inform future conservation management actions for the three guenon species of the Bijagós Archipelago in Guinea‐Bissau. Our results are potentially applicable to other widespread and genetically diverse taxa, now under increasing threats from anthropogenic activities.

### 4.1 Genome‐Wide Effects of Insularity

In all three guenon species considered here, island populations were genetically differentiated from the mainland populations. The timing of the divergence between insular and mainland populations varied between species but has generally happened before the start of the LGP (> 100,000 YA), according to bPSMC and bpp analyses. Since this time period, the island populations have experienced a steadier decline in the estimated *N*_e_ than their mainland counterparts. Nonetheless, *N*_e_ estimates were not alarmingly low for any of the insular populations (*N*_e_ > 2300; Frankham et al. 2014; Pérez‐Pereira et al. 2022). The observed lower genome‐wide heterozygosity and higher *F*_ROH_ in the insular populations of all three species compared to mainland populations support the long‐term reduction of the *N*_e_ as suggested by the demographic analyses. The genome‐wide effects of population bottlenecks associated with island colonisation could be further exacerbated in archipelagos, where sequential colonisation events from the initial founding populations are likely (Allendorf et al. 2013; Martin et al. 2023). In our study species, this effect can potentially be observed in the spot‐nosed monkey individual from Caravela, the outermost island of the Bijagós Archipelago. This individual has lower genome‐wide diversity and higher *F*_ROH_ compared to the Canhabaque population.

The timing of divergence between the insular and mainland populations as estimated by the bPSMC and BPP analyses is unexpectedly old considering the proposed timing of isolation of the Bijagós Archipelago during the Holocene (ca 12,000–6000 YA; Alves 2007). Previous work has shown that *N*_e_ fluctuations from coalescent‐based methods can reflect true changes in *N*_e_, cessation of panmixia, or both (Mazet et al. 2016). Indeed, the timing of the divergence of the populations coincides with cycles of dryer and wetter climate and, consequently, repeated retraction and expansion of forests across sub‐Saharan Africa (Hoag and Svenning 2017)—a known promoter of changes in connectivity in forest dwelling species such as primates (van der Valk et al. 2024). Thus, it is likely that the reported divergence of the *N*_e_ trajectories of bPSMC and BPP reflects a scenario of the disruption of panmixia/population structure arising prior to the colonisation of the archipelago. Moreover, the mainland populations included here may not represent the most closely related source populations, particularly for the spot‐nosed monkey, whose presence has not been reported from mainland Guinea‐Bissau for over 30 years (Bersacola et al. 2018, 2022; Gippoliti and Dell'Omo 2003; Colmonero‐Costeira, personal communication). On the other hand, the more recent (3000 YA) bottleneck‐like effect detected in GONE for the spot‐nosed monkeys of Canhabaque Island could represent the island colonisation event of this population. However, our results should be interpreted with caution as the reliability of point estimates of the analysis decreases after 100 generations in the past (~1100 YA; Santiago et al. 2020).

A recurring question in conservation genomics regarding the fate of small and inbred populations, such as those inhabiting insular systems, is whether genetic load is accumulating after a demographic bottleneck or if genetic purging is effective in decreasing the genetic load of highly deleterious mutations, thus preventing mutational meltdown. The decrease in the frequency of LoF variants following the conversion of masked load (i.e., heterozygous states of partially recessive deleterious mutations) into realised load (i.e., homozygous states) is often the main diagnostic feature of purging in bottlenecked populations (Dussex et al. 2021; Grossen et al. 2020; Khan et al. 2021; van der Valk et al. 2024; von Seth et al. 2022; Xue et al. 2015). Although a higher proportion of homozygous LoF variants was found in the insular guenon populations studied here, similar patterns were present in missense and neutral variants. Furthermore, after standardising the genetic load to the number of segregating derived sites (i.e., relative abundance of derived alleles), we observed that insular and mainland individuals have similar numbers of deleterious alleles across all variant categories, which is expected under genetic drift alone (Dussex et al. 2023; Smeds and Ellegren 2023).

Another commonly reported genomic consequence of prolonged periods of small *N*_e_ is the accumulation of mildly deleterious (e.g., missense) mutations (Dussex et al. 2023). Since drift diminishes the effectiveness of purifying selection, *de novo* mildly deleterious mutations are more likely to drift to higher frequencies in small populations (Dussex et al. 2023). We do not observe such a pattern in the insular guenon populations, suggesting that the effectiveness of purifying selection against deleterious derived alleles has been retained overall (Dussex et al. 2023). We argue that the insular guenon populations of the Bijagós Archipelago lack the typical genomic features of small, inbred populations. Our results suggest that the increased inbreeding and loss of genetic diversity in the insular populations have, so far, not led to increased purging of highly deleterious variants nor accumulation of genetic load (Dussex et al. 2023; Robinson et al. 2016; Taylor et al. 2024).

Several guenon species have been successful in establishing insular populations following human‐assisted introduction (e.g., green monkeys in Cape Verde (Almeida et al. 2024; Hazevoet and Masseti 2011) and multiple Caribbean Islands (Denham and Denham 1981), mona monkeys, *Cercopithecus mona*, in São Tomé and Príncipe and Grenada (Glenn and Bensen 2013; Horsburgh et al. 2003)). Based on historical records, these populations are thought to have been founded by relatively small numbers of individuals and the populations have increased to thousands of individuals within 300–500 years. While most of these populations remain poorly studied, there are no reports suggesting the presence of deleterious inbreeding effects on the islands when compared to mainland Africa (e.g., Grenada mona monkeys; Glenn and Bensen 2013). The success of guenon colonisation and potential avoidance of inbreeding effects could be attributed to a high ecological and behavioural flexibility and the relatively beneficial island ecosystems (e.g., decreased interspecific competition; Glenn and Bensen 2013) and overall high genetic diversity (Jensen et al. 2023; Kuderna et al. 2023).

Finally, we observed differentially fixed variation between insular and mainland populations. Some of the affected genes were present in two or three species and encoded for metabolic and developmental processes, which included cellular proliferation and organ size. While the specific phenotypic expression of these genes is unknown, these functional categories are among those commonly associated with the ‘island syndrome’ in vertebrate species (Nolte et al. 2020; Payseur and Jing 2021; Sendell‐Price et al. 2020). As populations are exposed to novel ecological challenges in insular ecosystems, which include changes in inter‐specific competition and predation dynamics, and access to novel food items, selection pressures acting in insular and source populations are likely to differ in several aspects (Welles and Dlugosch 2019). Common adaptations to the same insular ecosystem are a possible mechanism behind the overlapping gene sets across the three species (Payseur and Jing 2021; Welles and Dlugosch 2019). However, since our results suggest that genetic drift is likely the predominant evolutionary mechanism differentiating insular and mainland populations, an alternative explanation is that these genes are under relaxed selection on the islands. The relaxation of selection in genes linked to increased fitness in the mainland populations would allow drift to fix non‐synonymous variants that may be otherwise deleterious on the mainland (Cui et al. 2021; Wang et al. 2023). We stress, however, that these results should be interpreted with caution as differences in phenotypic traits between the guenon populations of the Bijagós Archipelago and the mainland populations have never been studied. Further the current mapping to a distant reference genome (rhesus macaque) and unbalanced sample sizes prevents an unbiased assessment of differentially fixed non‐synonymous variation, as neutrally evolving variation could be wrongly annotated as protein coding variation (e.g., pseudogenes).

#### 4.1.1 Importance of Insular Populations for the Conservation of Genetically Diverse Species

Even though guenon populations of the Bijagós Archipelago are thought to be more threatened than their mainland counterparts—due to the overall loss of genetic diversity, *N*_e_, and inbreeding associated with insularity—this study did not find increased accumulation of deleterious variation on the islands. However, the increased inbreeding and genetic drift on the islands resulted in higher levels of realised load, which may affect population fitness negatively. We found fixed LoF variants in genes involved in immune and reproductive functions in the insular populations of the three guenon species, although their fitness effects are unknown.

It is important to highlight that natural processes are not the only factor determining individual survival of these primates. Habitat degradation and harvesting of individuals for meat consumption are current conservation threats, which may increase their risk of extinction (Colmonero‐Costeira et al. 2023). Indeed, the insular populations of the three species show a significantly higher fraction of their genome in long runs of homozygosity, which likely arose due to the mating of closely related individuals in the last 1200 years after the arrival of humans on the archipelago. In particular, the illegal harvesting for the commercial meat trade has been described recently and is thought to be an increasing practice on the islands (Colmonero‐Costeira et al. 2023). In the absence of immediate threats from genetic factors, the long‐term conservation of these insular populations should be promoted mainly by reducing their immediate threats and safeguarding against additional loss of diversity by anthropogenic factors. For Guinea‐Bissau in particular, the insular guenon populations are of high conservation interest, particularly the spot‐nosed monkeys. We show that these relict populations are not genetically depauperate and could potentially act as reservoirs for the recently extinct (or very rare) mainland populations, creating the opportunity for future re‐introduction of the species in mainland Guinea‐Bissau.

Recent studies have proposed that small and threatened populations that underwent extensive purging are expected to be less sensitive to the effects of additional bottlenecks and can tolerate smaller *N*_e_ and still avoid severe inbreeding depression and extinction due to genetic factors (Caballero et al. 2016). However, not much is known about the response of genetically diverse species to demographic bottlenecks, such as the insular guenons studied here. Akin to this system, high realised genetic load in the absence of purging following population bottlenecks has been reported in other widespread and diverse species, such as the North American caribou, *Rangifer tarandus* (Taylor et al. 2024), and the Channel Island fox, *Urocyon littoralis* (Robinson et al. 2016). In these populations the interplay between the increasing anthropogenic threats and the dynamics of deleterious variation following demographic declines remains poorly studied and ought to be further investigated in order to pre‐emptively promote their conservation.

## Author Contributions

**I. Colmonero‐Costeira, A. Jensen, K. Guschanski, I. M. Russo, M. W. Bruford** and **M. J. Ferreira da Silva:** conceptualization. **I. Colmonero‐Costeira** and **A. Jensen:** methodology and analyses. **S. L. Djaló, N. Fernandes, I. Colmonero‐Costeira** and **M. J. Ferreira da Silva:** sample acquisition. **M. J. Ferreira da Silva, K. K.‐H. Farh, L. K. K. Kuderna, J. Rogers** and **T. Marques‐Bonet:** funding acquisition. **K. K.‐H. Farh, L. K. K. Kuderna, J. Rogers** and **T. Marques‐Bonet:** data production and processing. **I. Colmonero‐Costeira:** writing – original draft. All authors: writing – review and editing.

## Ethics Statement

All collected tissue samples used in this work were provided voluntarily and free of charge by local hunters and/traders. Informed consent to participate was obtained before sample collection. The providers of samples remained anonymous to local agencies and law enforcers. All sampling was carried out with the approval of the National Institute for Biodiversity and Protected Areas (IBAP). Direcção Geral das Florestas e Fauna (DGFF) authorised transportation and issued CITES export permits for tissue samples. Instituto da Conservação da Natureza e Florestas (ICNF, Portugal) issued the CITES importation permits to Portugal ( *Cercopithecus petaurista* —No. 18PTLX00592I and 23PTLX00512I, *Cercopithecus campbelli—*No. 18PTLX00590I, *Chlorocebus sabaeus* —No./No 18PTLX00586I). The Nagoya protocol was not in place in Guinea‐Bissau at the time of sampling. However, we acknowledged the work of local researchers by including them in research decisions, dissemination of results and as co‐authors of this manuscript. Results have been shared with the local governmental agencies through regular meetings and progress reports. Results of this work will be disseminated to the local communities through meetings and communications in local media.

## Conflicts of Interest

L.F.K.K. and K.‐H.K.F. are employees of Illumina Inc.

## Supporting information


**Figure S1.** Spot‐nosed monkey ( *Cercopithecus petaurista* ) haplotype network based on whole mitochondrial sequences. Haplotype size is proportional to the number of samples and are coloured according to their sampling location in Guinea‐Bissau. There are no shared haplotypes between sampling locations. Note that the spot‐nosed monkey Phase_1 mainland sample is of unknown origin (Kuderna et al. 2023) but likely belongs to the Eastern spot‐nosed monkey subspecies ( *Cercopithecus petaurista petaurista* ). The number of mutational steps between haplotypes is annotated on the branches. Illustrations copyright 2022 Stephen D. Nash/IUCN SSC Primate Specialist Group. Used with permission.
**Figure S2.** Spot‐nosed monkey ( *Cercopithecus petaurista* ) haplotype network based on whole mitochondrial sequences. Mitochondrial genomes publicly available in GenBank (https://www.ncbi.nlm.nih.gov/genbank/) were included and sampling locations were extracted from Guschanski et al. 2013. Haplotype size is proportional to the number of samples and are coloured according to their sampling location in Guinea‐Bissau. There are no shared haplotypes between sampling locations. Note that the spot‐nosed monkey Phase_1 mainland sample is of unknown origin (Kuderna et al. 2023) but likely belongs to the Eastern spot‐nosed monkey subspecies ( *Cercopithecus petaurista petaurista* ). The number of mutational steps between haplotypes is annotated on the branches. Illustrations copyright 2022 Stephen D. Nash/IUCN SSC Primate Specialist Group. Used with permission.
**Figure S3.** Campbell’s monkey ( *Cercopithecus campbelli* ) haplotype network based on whole mitochondrial sequences. Haplotype size is proportional to the number of samples and are coloured according to their sampling location in Guinea‐Bissau. There are no shared haplotypes between sampling locations. The number of mutational steps between haplotypes is annotated on the branches. Illustrations copyright 2022 Stephen D. Nash/IUCN SSC Primate Specialist Group. Used with permission.
**Figure S4.** Green monkey ( *Chlorocebus sabaeus* ) haplotype network based on whole mitochondrial sequences. Haplotype size is proportional to the number of samples and are coloured according to their sampling location in Guinea‐Bissau. There are no shared haplotypes between sampling locations. The number of mutational steps between haplotypes is annotated on the branches. Illustrations copyright 2022 Stephen D. Nash/IUCN SSC Primate Specialist Group. Used with permission.
**Figure S5.** Beta‐PSMC analysis for all the spot‐nosed monkey ( *Cercopithecus petaurista* ; left), Campbell’s monkey ( *Cercopithecus campbelli* ; middle) and green monkey ( *Chlorocebus sabaeus* ; right) genomes. Each line represents a distinct genome and are coloured according to the sampling location. Illustrations copyright 2022 Stephen D. Nash/IUCN SSC Primate Specialist Group. Used with permission.
**Figure S6.** Demographic history of spot‐nosed monkey ( *Cercopithecus petaurista* ), Campbell’s monkey ( *Cercopithecus campbelli* ; middle) and green monkey ( *Chlorocebus sabaeus* ) populations as estimated with BPP. Widths of the branches correspond to inferred effective population sizes (*N*_e_). Each population is coloured according to the sampling location. Due to the lack of differentiation, green monkeys from Ganogo and Orango islands were considered as one population in these analyses (OIG).
**Figure S7.** Recent demographic history of spot‐nosed monkey population of Canhabaque Island ( *Cercopithecus petaurista* ) as estimated with GONE. Solid lines represent the median effective population size across 20 independent runs. Dashed lines represent the 95% confidence interval.
**Figure S8.** Venn diagram of illustrating the number of protein‐coding genes containing differentially fixed variants overlapping in the spot‐nosed monkey ( *Cercopithecus petaurista* : top left), the Campbell’s monkey ( *Cercopithecus campbelli* ; top right) and the green monkey ( *Chlorocebus sabaeus* ; bottom). Each line represents a distinct genome and are coloured according to the sampling location.

**Table S1.** Number of segregating sites per category of mutational effects on protein‐coding genes for the three guenon species.
**Supplementary Information S1.** Sample information and autosome mapping coverage.
**Supplementary Information S2.** List of genes containing LoF variants that have become fixed in the homozygous state in insular populations.
**Supplementary Information S3.** GO enrichment analysis of genes that contained differentially fixed variants in at least two of the studied guenon species.

## Data Availability

The sequencing data used in this project are available on the European nucleotide archive (https://www.ebi.ac.uk/ena), under project accession PRJEB90502. Custom scripts used for data analyses are available at https://github.com/Colmonero‐CI/Guenons_POPGENOM.
