# Supplementary material for: Genomic Signatures of Island Colonisation in Highly Diverse Primates
Source: Mol Ecol. 2025 Jul 9;34(23):e17815. doi: 10.1111/mec.17815 (PMC12684299; doi:10.1111/mec.17815)
Supplement: Supplementary file 1 — Figure S1. Spot‐nosed monkey ( Cercopithecus petaurista ) haplotype network based on whole mitochondrial sequences. Haplotype size is proportional to the number of samples and are coloured according to their sampling location in Guinea‐Bissau. There are no shared haplotypes between sampling locations. Note that the spot‐nosed monkey Phase_1 mainland sample is of unknown origin (Kuderna et al. 2023) but likely belongs to the Eastern spot‐nosed monkey subspecies ( Cercopithecus petaurista petaurista ). The number of mutational steps between haplotypes is annotated on the branches. Illustrations copyright 2022 Stephen D. Nash/IUCN SSC Primate Specialist Group. Used with permission. Figure S2. Spot‐nosed monkey ( Cercopithecus petaurista ) haplotype network based on whole mitochondrial sequences. Mitochondrial genomes publicly available in GenBank (https://www.ncbi.nlm.nih.gov/genbank/) were included and sampling locations were extracted from Guschanski et al. 2013. Haplotype size is proportional to the number of samples and are coloured according to their sampling location in Guinea‐Bissau. There are no shared haplotypes between sampling locations. Note that the spot‐nosed monkey Phase_1 mainland sample is of unknown origin (Kuderna et al. 2023) but likely belongs to the Eastern spot‐nosed monkey subspecies ( Cercopithecus petaurista petaurista ). The number of mutational steps between haplotypes is annotated on the branches. Illustrations copyright 2022 Stephen D. Nash/IUCN SSC Primate Specialist Group. Used with permission. Figure S3. Campbell’s monkey ( Cercopithecus campbelli ) haplotype network based on whole mitochondrial sequences. Haplotype size is proportional to the number of samples and are coloured according to their sampling location in Guinea‐Bissau. There are no shared haplotypes between sampling locations. The number of mutational steps between haplotypes is annotated on the branches. Illustrations copyright 2022 Stephen D. Nash/IUCN SSC Primate Special [file MEC-34-e17815-s001.pdf]

MOLECULAR ECOLOGY

Supplemental Information for:

Genomic signatures of island colonization in highly diverse primates

Colmonero-Costeira I., Guschanski K., Djaló, M.L., Fernandes, N., Camará, T., Farh K.K.-H., Kuderna, L., Rogers J., Marques-Bonet, T., Bruford M.W., Russo I.M., Jensen A., Ferreira da Silva M.J.

Table of Contents:

**Fig. S1-S4. Haplotype networks based on whole mitochondrial sequences.**	Page 2-5
**Fig. S5. Beta-PSMC analysis for all the spot-nosed monkey, Campbell's monkey and green monkey genomes.**	Page 6
**Fig. S6. Demographic history of spot-nosed monkey, Campbell's monkey, and green monkey populations as estimated with BPP.**	Page 7
**Fig. S7. Recent demographic history of spot-nosed monkey population of Canhabaque Island as estimated with GONE.**	Page 8
**Fig. S8. Venn diagram of illustrating the number of protein-coding genes containing differentially fixed variants overlapping in the three guenon species.**	Page 9
**Table S1. Number of segregating sites per category of mutational effects on protein-coding genes for the three guenon species.**	Page 10

MOLECULAR ECOLOGY

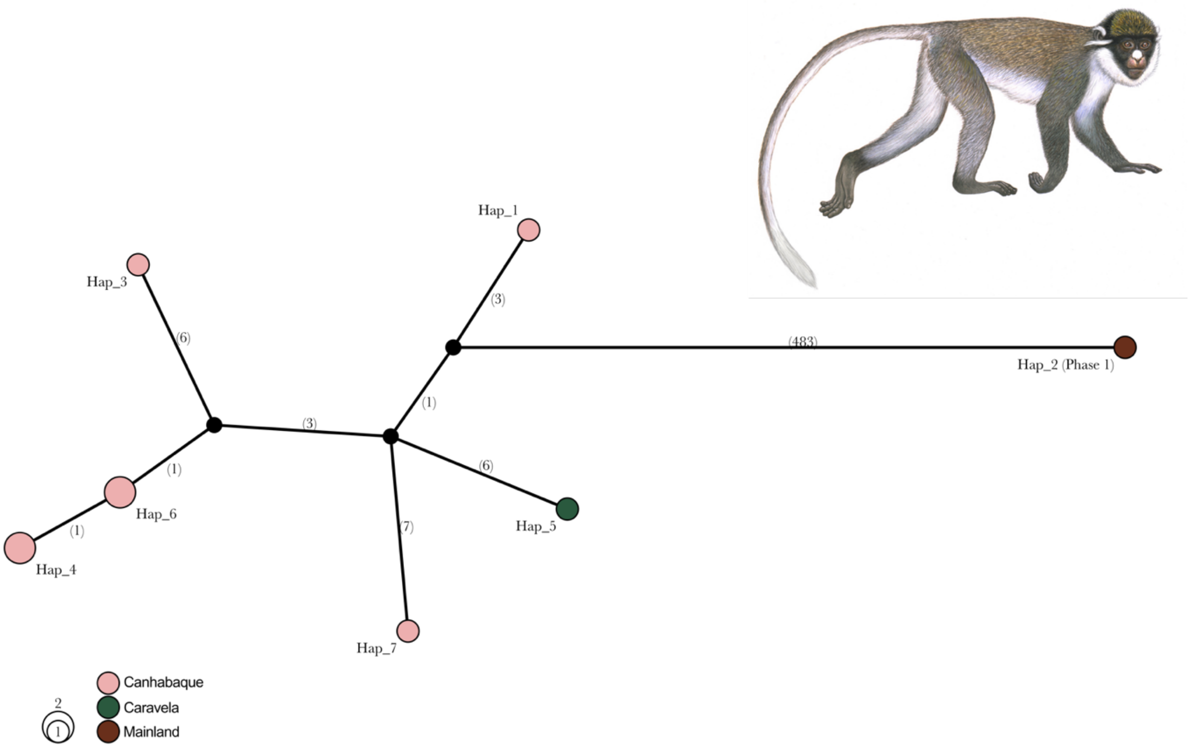

Fig. S1. Spot-nosed monkey (*Cercopithecus petaurista*) haplotype network based on whole mitochondrial sequences. Haplotype size is proportional to the number of samples and are coloured according to their sampling location in Guinea-Bissau. There are no shared haplotypes between sampling locations. Note that the spot-nosed monkey Phase_1 mainland sample is of unknown origin (Kuderna et al., 2023) but likely belongs to the Eastern spot-nosed monkey subspecies (*Cercopithecus petaurista petaurista*). The number of mutational steps between haplotypes is annotated on the branches. Illustrations copyright 2022 Stephen D. Nash / IUCN SSC Primate Specialist Group. Used with permission.

MOLECULAR ECOLOGY

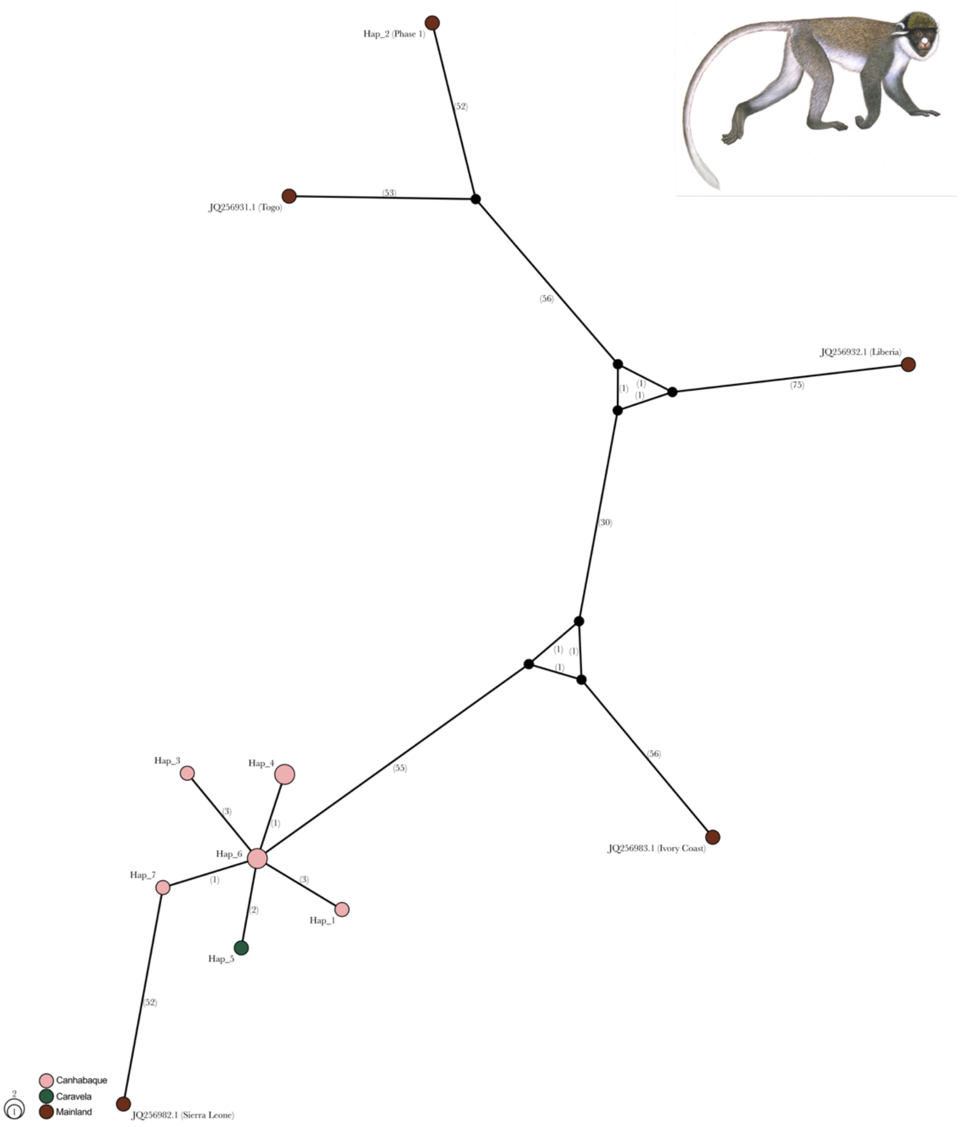

Fig. S2. Spot-nosed monkey (*Cercopithecus petaurista*) haplotype network based on whole mitochondrial sequences. Mitochondrial genomes publicly available in GenBank (https://www.ncbi.nlm.nih.gov/genbank/) were included and sampling locations were extracted from Guschanski et al., 2013. Haplotype size is proportional to the number of samples and are coloured according to their sampling location in Guinea-Bissau. There are no shared haplotypes between sampling locations. Note that the spot-nosed monkey Phase_1 mainland sample is of unknow origin (Kuderna et al., 2023) but likely belongs to the Eastern spot-nosed monkey subspecies (*Cercopithecus petaurista petaurista*). The number of mutational steps between haplotypes is annotated on the branches. Illustrations copyright 2022 Stephen D. Nash / IUCN SSC Primate Specialist Group. Used with permission.

MOLECULAR ECOLOGY

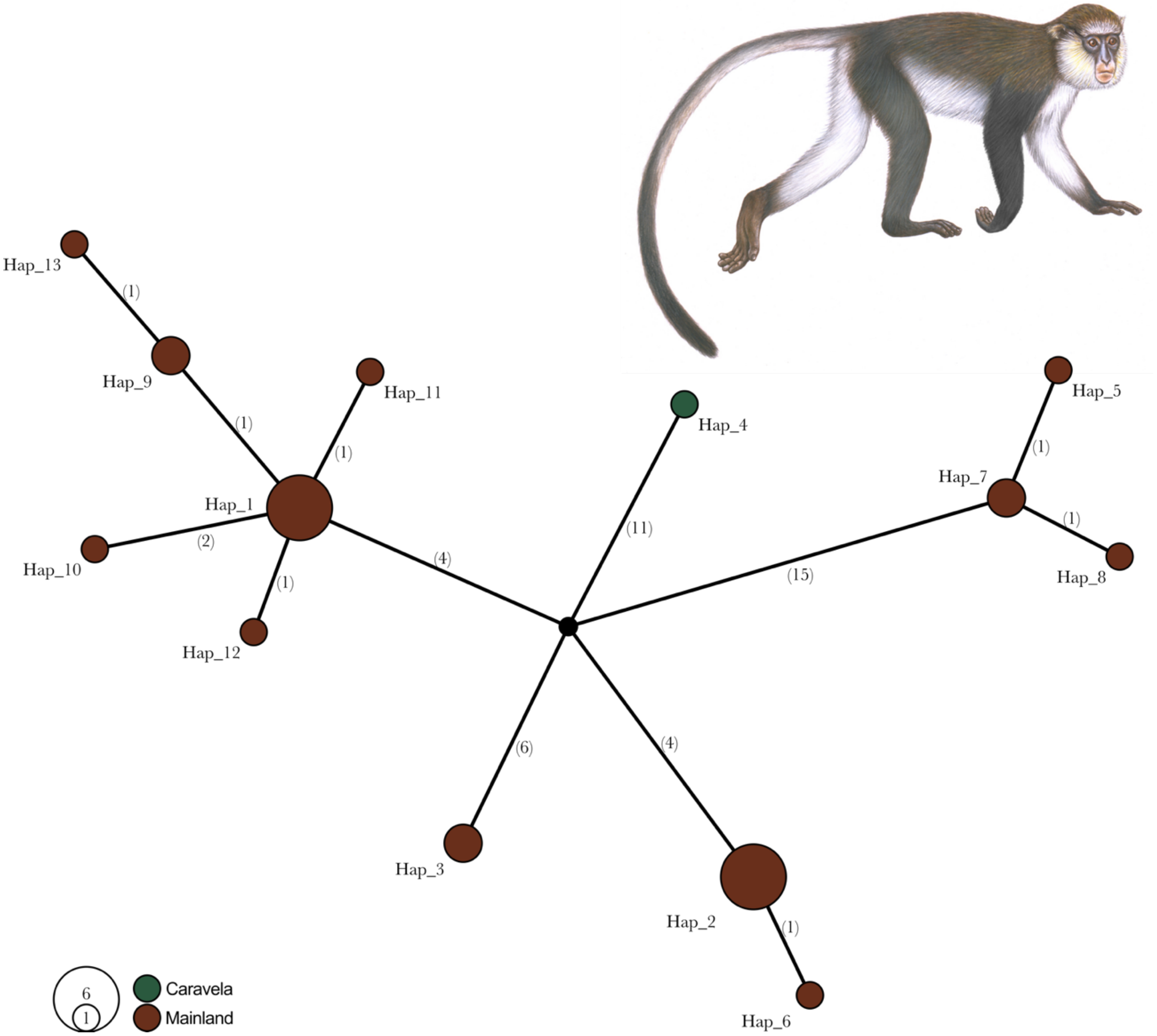

Fig. S3. Campbell's monkey (*Cercopithecus campbelli*) haplotype network based on whole mitochondrial sequences. Haplotype size is proportional to the number of samples and are coloured according to their sampling location in Guinea-Bissau. There are no shared haplotypes between sampling locations. The number of mutational steps between haplotypes is annotated on the branches. Illustrations copyright 2022 Stephen D. Nash / IUCN SSC Primate Specialist Group. Used with permission.

MOLECULAR ECOLOGY

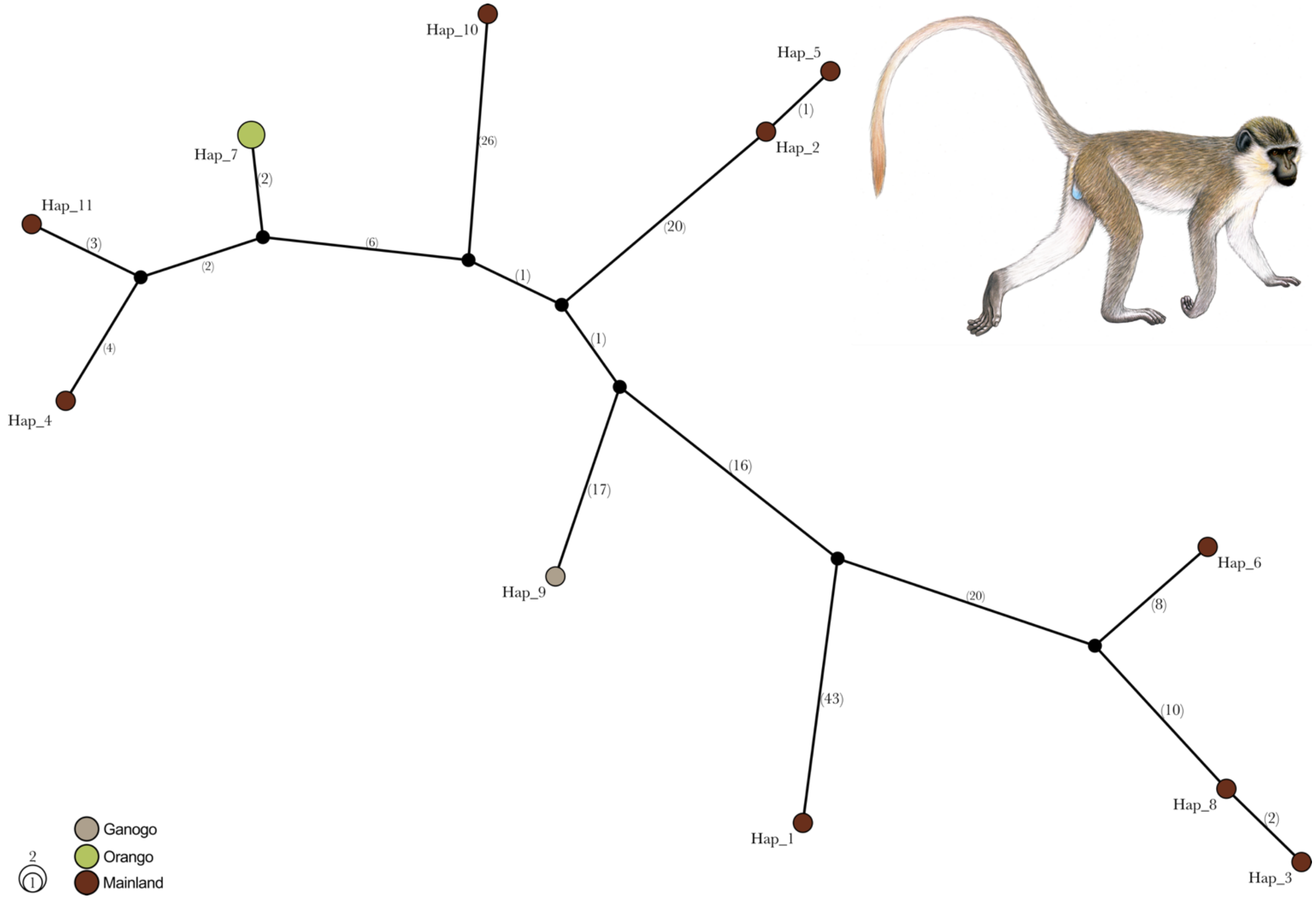

Figure S4. Green monkey (*Chlorocebus sabaeus*) haplotype network based on whole mitochondrial sequences. Haplotype size is proportional to the number of samples and are coloured according to their sampling location in Guinea-Bissau. There are no shared haplotypes between sampling locations. The number of mutational steps between haplotypes is annotated on the branches. Illustrations copyright 2022 Stephen D. Nash / IUCN SSC Primate Specialist Group. Used with permission.

MOLECULAR ECOLOGY

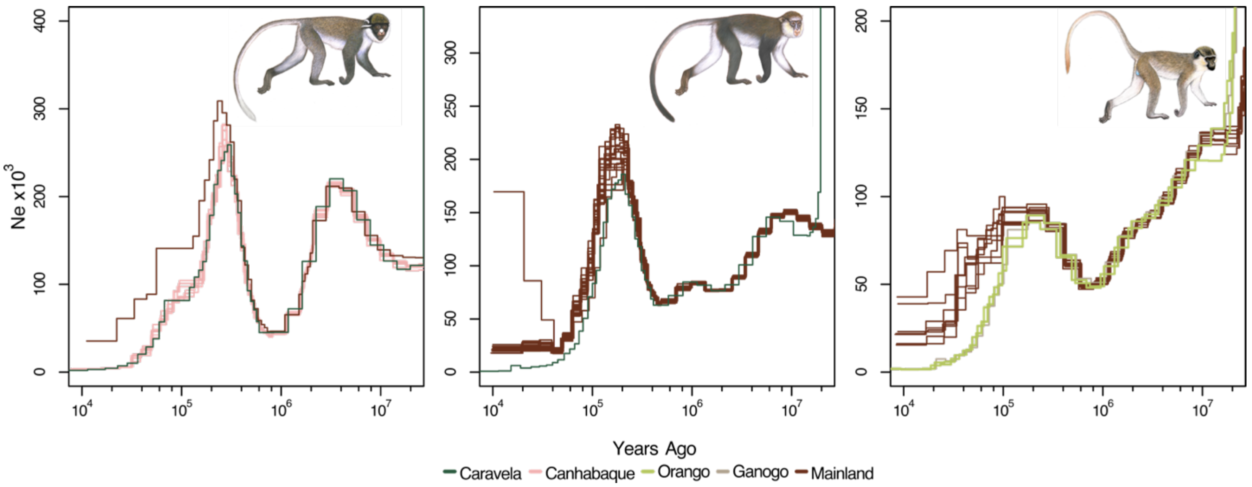

Fig. S5. Beta-PSMC analysis for all the spot-nosed monkey (*Cercopithecus petaurista*; left), Campbell's monkey (*Cercopithecus campbelli*; middle), and green monkey (*Chlorocebus sabaeus*; right) genomes. Each line represents a distinct genome and are coloured according to the sampling location. Illustrations copyright 2022 Stephen D. Nash / IUCN SSC Primate Specialist Group. Used with permission.

MOLECULAR ECOLOGY

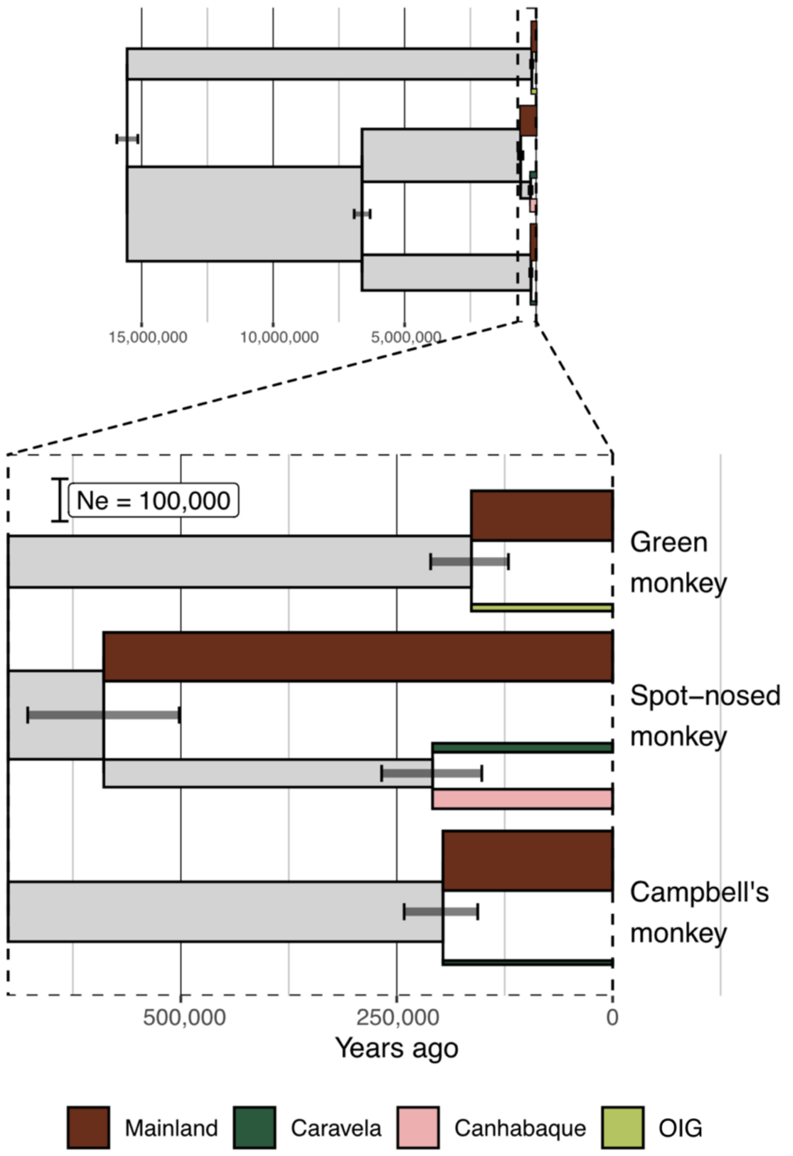

Fig. S6. Demographic history of spot-nosed monkey (*Cercopithecus petaurista*), Campbell's monkey (*Cercopithecus campbelli*; middle), and green monkey (*Chlorocebus sabaeus*) populations as estimated with BPP. Widths of the branches correspond to inferred effective population sizes (N_e). Each population is coloured according to the sampling location. Due to the lack of differentiation, green monkeys from Ganogo and Orango islands were considered as one population in these analyses (OIG).

MOLECULAR ECOLOGY

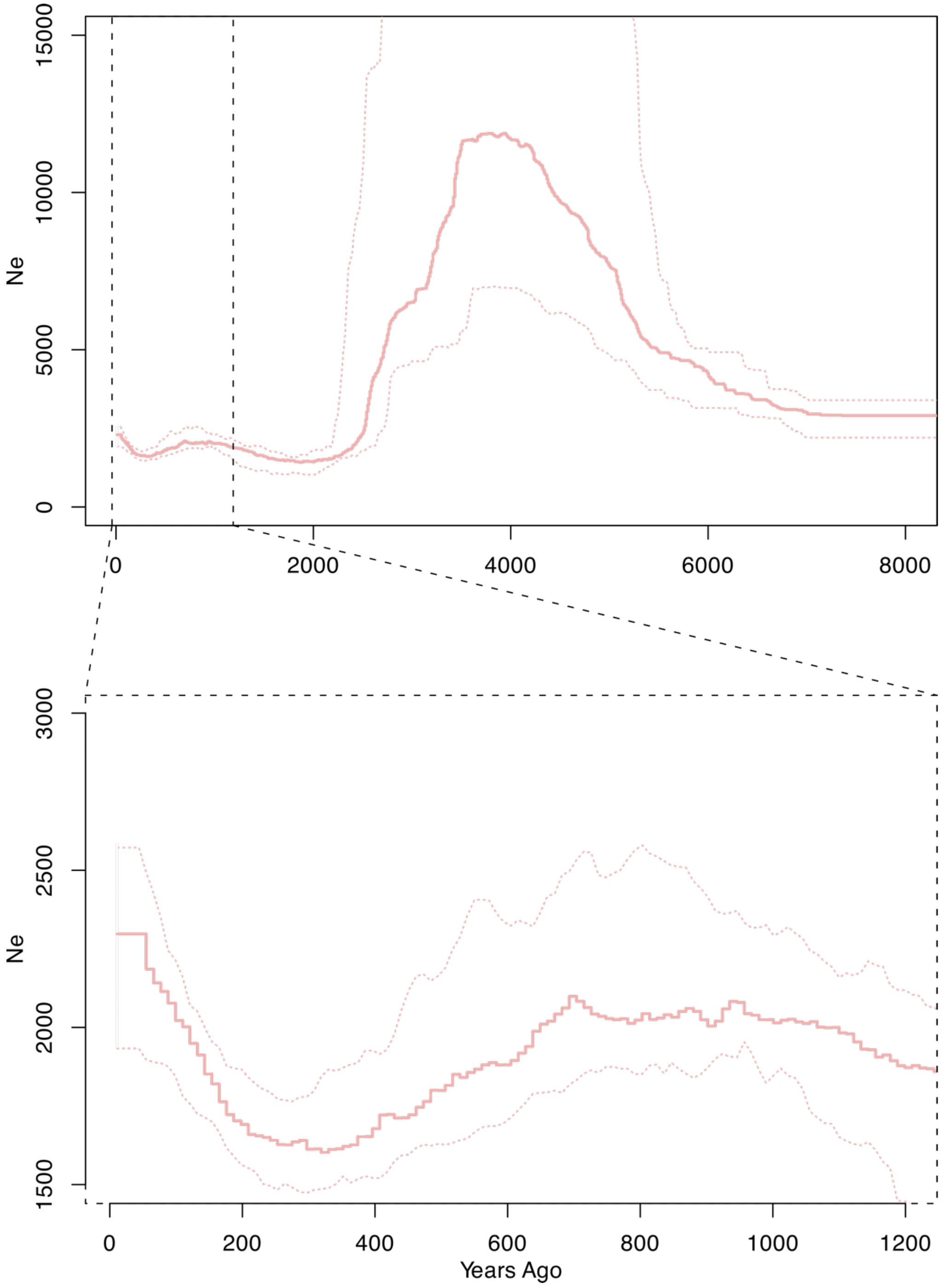

Fig. S7. Recent demographic history of spot-nosed monkey population of Canhabaque Island (*Cercopithecus petaurista*) as estimated with GONE. Solid lines represent the median effective population size across 20 independent runs. Dashed lines represent the 95% confidence interval.

MOLECULAR ECOLOGY

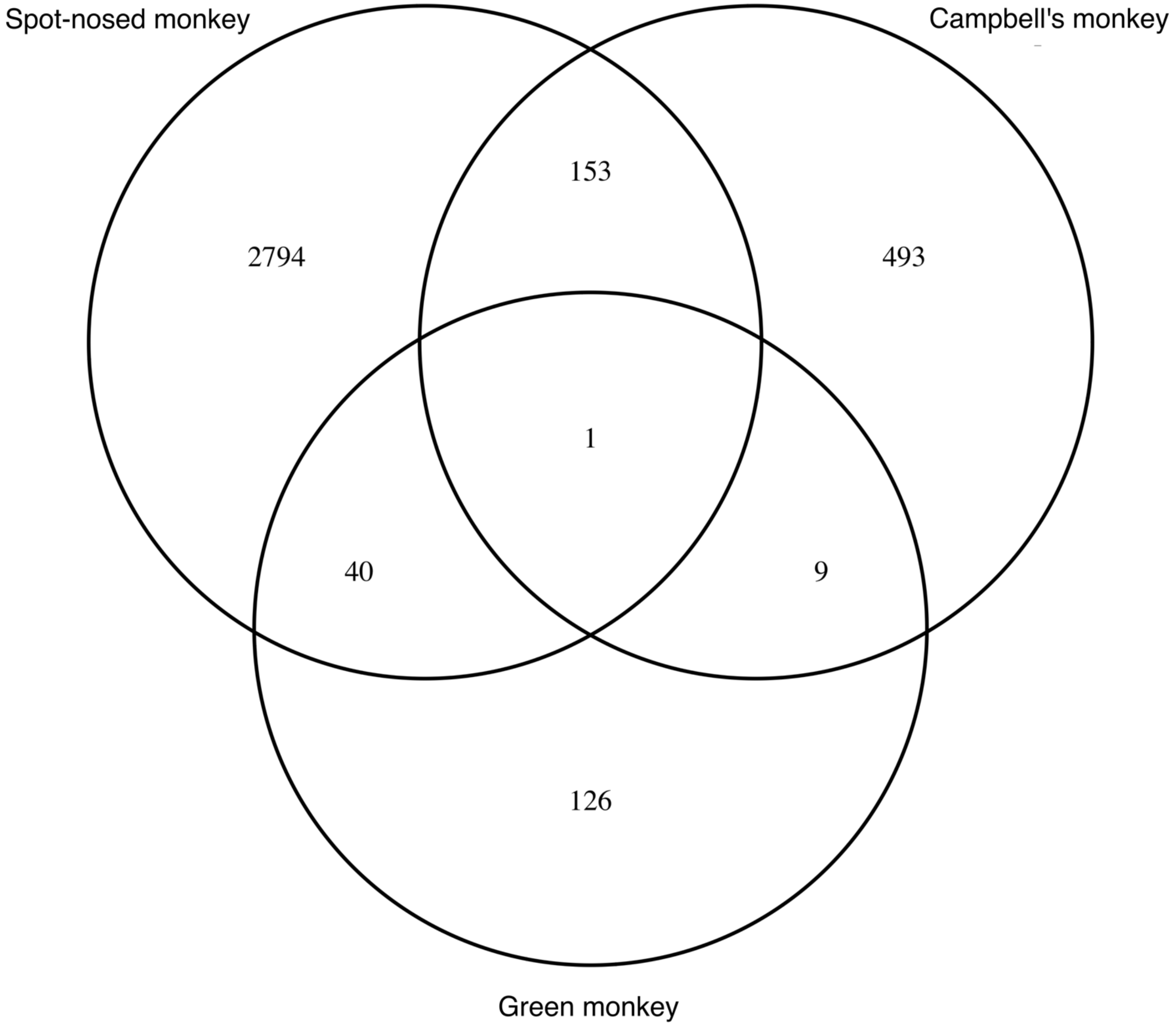

Fig. S8. Venn diagram illustrating the number of protein-coding genes containing differentially fixed variants overlapping in the spot-nosed monkey (*Cercopithecus petaurista*: top left), the Campbell's monkey (*Cercopithecus campbelli*; top right) and the green monkey (*Chlorocebus sabaeus*; bottom). Each line represents a distinct genome and are coloured according to the sampling location. Illustrations copyright 2022 Stephen D. Nash / IUCN SSC Primate Specialist Group. Used with permission.

MOLECULAR ECOLOGY

Table S1. Number of segregating sites per category of mutational effects on protein-coding genes for the three guenon species.

**Spot-nosed monkey**				
	Caravela	Canhabaque [†]	Mainland	Total[‡]
Synonymous	66,289	68,926±803	71,565	107,964
Tolerated missense (Ex)	29,216	30,181±347	31,165	50,921
Deleterious missense (Ex)	10,959	11,409±144	11,823	20,303
LoF	1,245	1,247±11	1,273	2,184
**Campbell's monkey**				
	Caravela		Mainland[†]	Total
Synonymous	66,531		71,804±673	126,229
Tolerated missense (Ex)	28,164		30,450±278	62,380
Deleterious missense (Ex)	10,857		11,853±129	25,872
LoF	1,195		1,255±18	2,633
**Green monkey**				
	OIG		Mainland[†]	Total
Synonymous	69,048±1807		71,424±1507	109,891
Tolerated missense (Ex)	30,027±757		31,346±639	54,552
Deleterious missense (Ex)	11,728±268		12,132±242	22,145
LoF	1,250±29		1,288±21	2,291

† for sampling locations with N > 3, values represent the mean ± SD
‡ total number of derived variants within species
EX missense mutations were classified as potentially deleterious or tolerated based on the experimental exchangeability score (Yampolsky et al., 2005).
